# Electrospun Orodispersible Films of Isoniazid for Pediatric Tuberculosis Treatment

**DOI:** 10.3390/pharmaceutics12050470

**Published:** 2020-05-21

**Authors:** Konstantina Chachlioutaki, Emmanouil K. Tzimtzimis, Dimitrios Tzetzis, Ming-Wei Chang, Zeeshan Ahmad, Christina Karavasili, Dimitrios G. Fatouros

**Affiliations:** 1 Laboratory of Pharmaceutical Technology, Department of Pharmaceutical Sciences, Aristotle University of Thessaloniki, GR-54124 Thessaloniki, Greece; kchachlio@auth.gr (K.C.); dfatouro@pharm.auth.gr (D.G.F.); 2 Digital Manufacturing and Materials Characterization Laboratory, School of Science and Technology, International Hellenic University, 14km Thessaloniki–N. Moudania, GR-57001 Thermi, Greece; m.tzimtzimis@ihu.edu.gr (E.K.T.); d.tzetzis@ihu.edu.gr (D.T.); 3 Nanotechnology and Integrated Bioengineering Centre, University of Ulster, Jordanstown Campus, Newtownabbey BT37 0QB, Northern Ireland, UK; m.chang@ulster.ac.uk; 4 Leicester School of Pharmacy, De Montfort University, Leicester LE1 9BH, UK; zahmad@dmu.ac.uk

**Keywords:** orodispersible films, electrospinning, pediatric drug delivery, isoniazid, tuberculosis

## Abstract

Child-appropriate dosage forms are critical in promoting adherence and effective pharmacotherapy in pediatric patients, especially those undergoing long-term treatment in low-resource settings. The present study aimed to develop orodispersible films (ODFs) for isoniazid administration to children exposed to tuberculosis. The ODFs were produced from the aqueous solutions of natural and semi-synthetic polymer blends using electrospinning. The spinning solutions and the resulting fibers were physicochemically characterized, and the disintegration time and isoniazid release from the ODFs were assessed in simulated salivary fluid. The ODFs comprised of nanofibers with adequate thermal stability and possible drug amorphization. Film disintegration occurred instantly upon contact with simulated salivary fluid within less than 15 s, and isoniazid release from the ODFs in the same medium followed after the disintegration profiles, achieving rapid and total drug release within less than 60 s. The ease of administration and favorable drug loading and release properties of the ODFs may provide a dosage form able to facilitate proper adherence to treatment within the pediatric patient population.

## 1. Introduction

Tuberculosis (TB) is an infectious disease ranked among the top ten causes of death worldwide. The pediatric population represents ca. 6% of the annual global TB burden, resulting in the deaths of up to 80,000 HIV-uninfected children every year in many TB endemic countries [1]. Children exposed to *Mycobacterium tuberculosis* infections are at increased risk of developing TB, especially in settings with high prevalence of the disease, and are therefore a primary target group in immediate need of preventive treatment. Chemoprophylaxis with isoniazid (ISO) has proven highly effective in mitigating the risk of developing active TB [2]. According to the World Health Organization’s (WHO) recommendations, isoniazid preventive therapy (IPT) should involve a daily dose of 5 mg/kg (maximum 300 mg) for young children (<5 years) [3], and that was lately amended to also include older (≥5 years) children without active TB for a period of at least six to nine months [4]. Shorter treatment regimens containing rifampicin have been also applied with similar efficacy, yet the highly prevalent adverse effects related to rifampicin treatment commonly result in treatment discontinuation [5].

IPT compliance is often challenging, as confirmed by several studies in low-resource settings [6], since 80% or greater medication adherence is a prerequisite for successful IPT. The combination of prolonged treatment on a daily basis with the existing dosage forms (tablets, syrup, intramuscular injection) results in poor uptake and adherence [7]. Pediatric patients commonly encounter difficulty in swallowing tablets, with caregivers resorting to crushing tablets and mixing with food to facilitate ingestion. These practices, however, may induce dosing errors resulting in reduced therapeutic efficacy or even toxicity. On the other hand, liquid dosage forms enable dosing flexibility, yet are confined by stability and taste-masking challenges [8], and by higher transportation and storage costs. Alongside the discomfort associated with injectable therapies, there is an urgency for age-appropriate formulation interventions that will enhance patient compliance and treatment adherence for effective pharmacotherapy. Flexible solid oral formulations, including multiparticulates—dispersible, effervescent, orally disintegrating and chewable tablets—have introduced an easy-to-swallow palatable alternative, though in some cases potable water may still be required for reconstitution of or co-administration with the dosage forms [9].

Recently, orodispersible films (ODFs) have attracted much interest for the oral administration of drugs within the pediatric population [10]. They are easy to administer without the need for water intake, overcoming any swallowing difficulties, since they instantly disintegrate in the mouth upon deposition on the tongue [11], they enable dose flexibility and personalization of dosage strength [12,13] and are easy to fabricate both in a hospital setting and on an industrial scale [12]. In a single site, open label trial in children aged six months old to five years old using placebo ODFs, a high degree of acceptability was observed among young children and their caregivers, with 79% of infant caregivers and 86% of preschool-children’s caregivers providing positive ratings for their children’s acceptance of the ODF [14].

The solvent casting has been the manufacturing method of choice for ODFs [15,16,17,18,19], yet several drawbacks have been associated with this method, including the necessity of using plasticizers, which may be subjected to scrutiny for their toxicity and environmental hazards, and for increasing the cost of the end product [20]. Electrospinning has provided a promising alternative to solvent casting, producing amorphous fibrous films with enhanced flexibility and plasticity, without using plasticizers, and significantly higher surface areas compared to cast films [21]. The choice of excipients and solvents in pediatric formulations is subjected to critical screening, avoiding those potentially toxic or unsuitable for children. Several studies have reported the fabrication of electrospun ODFs from synthetic polymers in non-aqueous solvents [22,23,24] or aqueous solutions [25], though there are only a few examples of natural polymers such as polysaccharides, proteins and cellulose derivatives electrospun to ODFs from aqueous solutions [26].

In this context, the aim of this work was to develop ODFs as age-appropriate dosage forms for the long-term chemoprophylaxis of the pediatric TB patient population with ISO. The ODFs were electrospun from aqueous blend solutions of natural (pullulan, pectin, sodium caseinate) and semi-synthetic (hydroxypropyl methylcellulose (HPMC)) polymers. Pullulan, a polysaccharide with film-forming properties, along with pectin, a heteropolysaccharide abundant in fruits and vegetables, and sodium caseinate, a milk protein, are all edible functional ingredients widely used in food and pharmaceutical applications, whereas HPMC is a multi-functional pharmaceutical excipient [27,28].

## 2. Materials and Methods

### 2.1. Materials

Isoniazid (ISO) was obtained from Fagron HELLAS (Athens, Greece). Pullulan (from *Aureobasidium pullulans*) was kindly provided from Falcon, Food Products Manufacturers Raw Materials (Athens, Greece). Hydroxypropyl methylcellulose (Hhpromellose 2910, (HPMC) viscosity: 4000 cP, 2% at H_2_O (20 °C)), sodium caseinate (casein sodium salt from bovine milk (NaCas)), pectin (pectin from apple, 50–75% degree of esterification) were purchased from Sigma-Aldrich (Steinheim, Germany). Distilled water was used for the preparation of solutions. All other chemicals were of analytical grade and used as purchased.

### 2.2. Preparation of Solutions for Electrospinning

Solutions were prepared by dissolving pullulan in distilled water at a final polymer concentration of 19% *w*/*v*. Pullulan/HPMC, pullulan/pectin and pullulan/NaCas blend solutions were prepared by dissolving HPMC, pectin and NaCas in the pullulan solutions at increasing concentrations (1–3% *w*/*v*), keeping the total polymer content constant at 19% *w*/*v*. For the drug loaded solutions, ISO was dissolved in the polymer blends (14% *w*/*w* of the polymer concentration). Solutions were magnetically stirred overnight at ambient conditions and vacuum degassed prior to electrospinning to remove entrapped air-bubbles.

### 2.3. Characterization of the Solutions

#### 2.3.1. Electrical Conductivity of the Spinning Solutions

The electrical conductivity of the blend solutions was evaluated using a conductivity meter (Fisherbrand™ accumet™ Basic AB30 Conductivity Meter, Fisher Scientific, Loughborough, UK). Measurements were performed in triplicate at ambient conditions. Prior to use the probe was calibrated by 0.01 M KCl standard solution of 1.41 mS/cm.

#### 2.3.2. Rheological Properties of the Spinning Solutions

The rheological properties of the spinning solutions were evaluated on a rotational Physica MCR 300 rheometer (Physica Messtechnik GmbH, Stuttgart, Germany) using the cone and plate geometry (plate diameter: 50 mm, cone angle: 1.0°, gap: 0.5 mm). The temperature was regulated at 25 °C by a Paar Physica circulating bath and a controlled Peltier system (TEZ 150P/MCR). The steady state flow properties of the blend solutions were assessed within their linear viscoelastic region (γ_ο_ = 0.1%) at the shear rate range between 0.1 and 1000 s^−1^. The apparent shear rate of the solutions during electrospinning process at the needle was calculated to be 0.16 s^−1^ according to Equation (1):

γ = 4Q/πR^3^ (1)

where the Q is the feed rate of solution (mL/h) and R is the diameter of the needle (gauge 18).

### 2.4. Electrospinning Process

The electrospinning set-up consisted of a high-voltage power supply; a syringe pump; a 5 mL plastic syringe with an 18-gauge stainless-steel needle, in which a positive charge was applied; and a grounded collector (Electrospinning System—Starter Kit, E-Fiber electrospinning system, SKE Research Equipment^®^ (Bollate, Italy)). Solutions were pumped at a flow rate of 0.5 mL/h and the applied voltage was set at 20 kV chosen based on preliminary optimization studies. The distance between the tip of the needle to the surface of the collector was set at 15 cm. Electrospinning was performed at room temperature 25.0 ± 0.2 °C and relative humidity of 45%. The electrospun mats were deposited on the grounded collector using aluminum foil as the collector substrate and were stored in a desiccator till further use.

### 2.5. Characterization of the Orodispersible Films (ODFs)

#### 2.5.1. Morphological Assessment of the ODFs

The morphology of the ODFs was assessed with scanning electron microscopy (Phenom ProX, ThermoFisher Scientific, Waltham, MA, USA). Samples were mounted onto double adhesive conductive carbon tabs (TED Pella, Redding, CA, USA) on an aluminum stub. The samples were gold-coated using an ion sputtering device (Quorum SC7620, East Sussex, UK) and scanned at an accelerating voltage of 15 kV. Fiber diameters were determined using Phenom FiberMetric—Fiber Analysis Software based on at least 100 fibers from three different images per sample (amplification 6000×).

#### 2.5.2. Fourier Transform Infrared (FTIR) Spectroscopy

FTIR spectroscopy was employed to identify potential interactions between the drug and the polymers. The FTIR spectra of the raw materials and the ODFs were recorded on a Shimadzu IR Prestige-21 spectrometer (Shimadzu, Kyoto, Japan) with a horizontal Golden-Gate MKII single reflection ATR system (Specac, Kent, UK) equipped with ZnSe lenses with the wavenumber range of between 4000 and 600 cm^−1^ at a resolution of 2 cm^−1^ after 64 scans.

#### 2.5.3. Differential Scanning Calorimetry (DSC) and Thermogravimetric Analysis (TGA)

Thermal analysis of the raw materials and the ODFs was performed on a 204 F1 Phoenix DSC apparatus (Netsch GmBH, Selb, Germany). Samples were sealed in aluminum crucibles with perforated lids. Analysis was performed at a heating rate of 10 °C/min, from 20 °C to 200 °C under a nitrogen flow of 50 mL/min. The thermal stability of the electrospun films was assessed on a Shimadzu TGA-50 instrument (Tokyo, Japan). Samples were heated on aluminum pans at a heating rate of 10 °C/min from 40 to 300 °C under a nitrogen purge of 50 mL/min.

### 2.6. High Performance Liquid Chromatography (HPLC)

ISO quantification was performed using a HPLC system consisting of a LC-10 AD VP pump, an autosampler model SIL-20A HT equipped with a 100 μL loop and a UV–vis detector model SPD-10A VP (Shimadzu, Kyoto, Japan). Analysis was performed on a Discovery^®^ C18 HPLC column (25 cm × 4.6 mm, 5 μm particle size) using acetonitrile and 50 mM potassium dihydrogen phosphate pH 3.5 (30:70 *v*/*v*) as the mobile phase at a flow rate of 0.6 mL/min. The injection volume was set at 30 μL and UV detection was performed at 262 nm. Calibration curves of ISO were linear (*r*^2^ > 0.999) in the concentration range of 50–200 μg/mL for all tested media.

### 2.7. Drug Content Quantification

Ten precisely weighed ODFs were cut from each individual sample and each was dissolved in 2 mL DI water. The solutions were centrifuged at 4500 rpm for 15 min and the supernatants were analyzed by HPLC. ISO content and encapsulation efficiency were calculated based on the following equations:

Drug content (% *w*/*w*) = 100 × W_drug in the fibers_/W_drug loaded fiber_ (2)

Encapsulation Efficiency (%) = 100 × W_drug in the fibers_/W_total drug_ (3)

### 2.8. Disintegration Time of the ODFs in Simulated Salivary Fluid (SSF)

The disintegration time of the ODFs was evaluated based on a previously reported method [29]. The ODFs were cut in 1.5 × 1.5 cm pieces and were placed in a Petri dish. Two mL of SSF [30] were instilled on the surface of the films and the procedure was recorded until their complete dissolution using a wide-angle camera (video and photo resolution 1080p and 12 MP). The disintegration time was estimated after converting the recorded videos to JPM images corresponding to specified time-points.

### 2.9. In Vitro Drug Release in Simulated Salivary Fluid

In vitro ISO release studies from the electrospun films were performed in SSF pH 7.0 at 37 °C under mild agitation (50 rpm). The ODFs (1.5 × 1.5 cm^2^) were weighed and immersed in glass vials filled with 5 mL of preheated SSF. At predetermined time intervals, 200 μL samples were withdrawn and quantified by HPLC after syringe filtration (0.45 μm PTFE). Experiments were repeated in triplicate.

### 2.10. Statistical Analysis

Data are expressed as mean values ± standard deviation. Statistical analysis was performed using student’s *t*-test and statistical significance was set at *p* < 0.05.

## 3. Results and Discussion

### 3.1. Physical Properties of the Spinning Solutions

The viscosity and electrical conductivity values of the spinning solutions are summarized in Table 1. Both properties have been shown to affect the morphologies of the resulting fibers. In particular, viscosity affects the extent of the polymers’ molecule chain entanglement within the solution; for highly viscous solutions the electrical charges may generate insufficient strength to stretch the solution into forming fibers, whereas for solutions of low viscosity the electrospinning jet may break up into droplets, with no fiber formation occurring [31].

**Figure 1:**
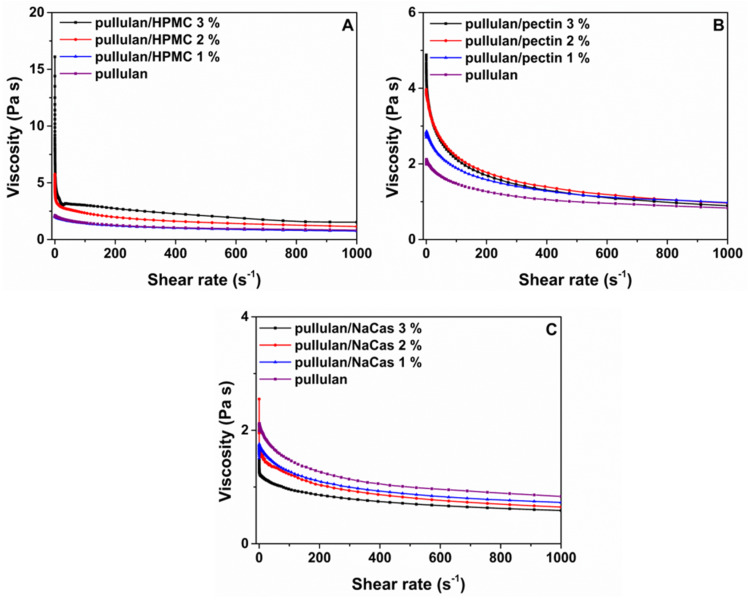
Viscosity as a function of shear rate for the pullulan solution and its blends with increasing concentrations of (**A**) HPMC, (**B**) pectin and (**C**) sodium caseinate.

As shown in Figure 1, increase in the applied shear rate decreased the flow resistance of all polymer solutions, indicative of pseudoplastic behavior. A major increase in the viscosity of pullulan solution was observed upon the addition of HPMC, with the effect being more significant for the highest HPMC concentrations (2% and 3%). In a similar manner, but to a lesser extent compared to HPMC, addition of pectin at 2% and 3% induced a slight increase in the viscosity of pullulan solution. This effect could be attributed to the molecular entanglements developed between the two polysaccharides, as previously described for pullulan-pectin blend solutions [32]. On the contrary, the effect of NaCas on the viscosity of the pullulan/NaCas blends was marginal, even for the highest concentrations tested (Figure 1C). This trend is clearly illustrated in the shear viscosity values obtained at 0.16 s^−1^ shear rate (Table 1).

The effects of HPMC, pectin and NaCas on the conductivities of their blend solutions with pullulan were also investigated (Table 1). The addition, all excipients resulted in a significant increase in conductivity for their blends compared to pullulan, which exhibited the lowest electrical conductivity value (65 ± 0.1 μS/cm), with the effect being more pronounced upon increasing their concentration from 1% *w*/*v* to 3% *w*/*v*.

### 3.2. Fiber Morphology

**Figure 2:**
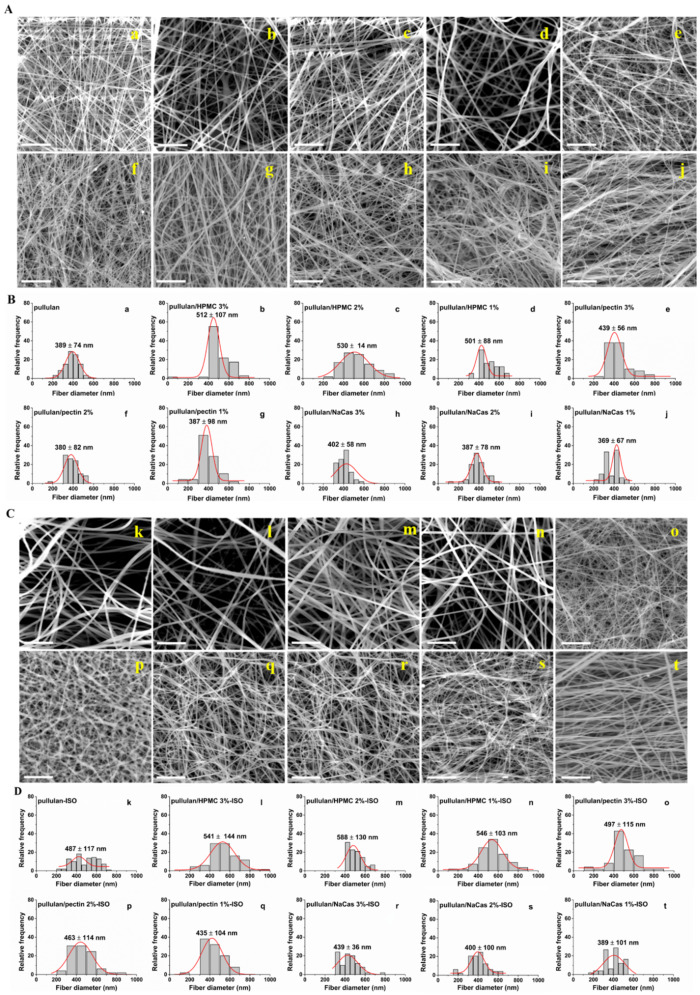
(**A**) and (**C**) SEM images of the ODF formulations containing (**a**) pullulan, (**b**) pullulan/HPMC 3%, (**c**) pullulan/HPMC 2%, (**d**) pullulan/HPMC 1%, (**e**) pullulan/pectin 3%, (**f**) pullulan/pectin 2%, (**g**) pullulan/pectin 1%, (**h**) pullulan/NaCas 3%, (**i**) pullulan/NaCas 2%, (**j**) pullulan/NaCas 1% and (**k**) pullulan-ISO, (**l**) pullulan/HPMC 3%-ISO, (**m**) pullulan/HPMC 2%-ISO, (**n**) pullulan/HPMC 1%-ISO, (**o**) pullulan/pectin 3%-ISO, (**p**) pullulan/pectin 2%-ISO, (**q**) pullulan/pectin 1%-ISO, (**r**) pullulan/NaCas 3%-ISO, (**s**) pullulan/NaCas 2%-ISO and (**t**) pullulan/NaCas 1%-ISO and their respective diameter distributions (**B**). (**a**–**j**) and (**D**). (**k**–**t**). Scale bar: 10 μm.

The complications arising from the inability of several biopolymers to be electrospun alone is being usually overcome with the aid of a carrier [32,33,34,35]. In the present study, pullulan was combined with a cellulose derivate, a polysaccharide and a protein to facilitate the fabrication of fast dissolving oral films for pediatric use. All prepared solutions of pullulan and pullulan blends with HPMC, pectin and NaCas were electrospun, producing fibrous films. The fiber morphologies of the plain and ISO-loaded ODFs are shown in Figure 2A,C, respectively, while the fiber diameter distributions of the plain and ISO-loaded ODFs are shown in Figure 2B,D, respectively.

All formulations demonstrated smooth fiber surface morphology and significant variations in their diameter distributions. A trend towards increase in the mean fiber diameter with increasing biopolymer concentration was evident for all ODF formulations. This may be associated with the concomitant viscosity increase of the blend solutions upon increasing biopolymers’ concentrations, as shown in Table 1. In the case of HPMC-containing ODFs, a larger fiber diameter and a boarder diameter distribution was observed, compared to the pectin and NaCas-containing ODFs. A similar trend was noted by Nazari et al. when the use of HPMC as co-polymer resulted in a boarder fiber diameter distribution [36]. The highest viscosity values obtained for the highest HPMC concentration could be attributed to the presence of the hydroxyl group in the HPMC molecule, which increases the water binding capacity and thus the viscosity of the blend solutions, resulting in fibers with larger diameters [37]. Furthermore, increase in the electrical conductivity has been shown to enhance the bending instability and induce jet lengthening and extensive stretching, thereby favoring the formation of thinner fibers [38,39], as in the case of the pectin and NaCas-containing ODFs.

The ISO-loaded nanofibers demonstrated a smooth surface morphology, indicating homogeneous drug encapsulation within the polymer matrices (Figure 2C). The drug-loaded nanofibers were larger in diameter compared to the plain polymeric nanofibers, which may be attributed to the addition of the drug increasing the concentration of the polymer solutions and hence leading to an enlargement in fiber diameter, as also previously reported [36].

The encapsulation efficiency (EE) and drug loading capacity (LC) values of the ISO-loaded ODFs are presented in Table 2. In general, electrospinning of all blended solutions contributed to high encapsulation efficiencies (≥ 90%).

### 3.3. Thermal Properties of the ODFs

**Figure 3:**
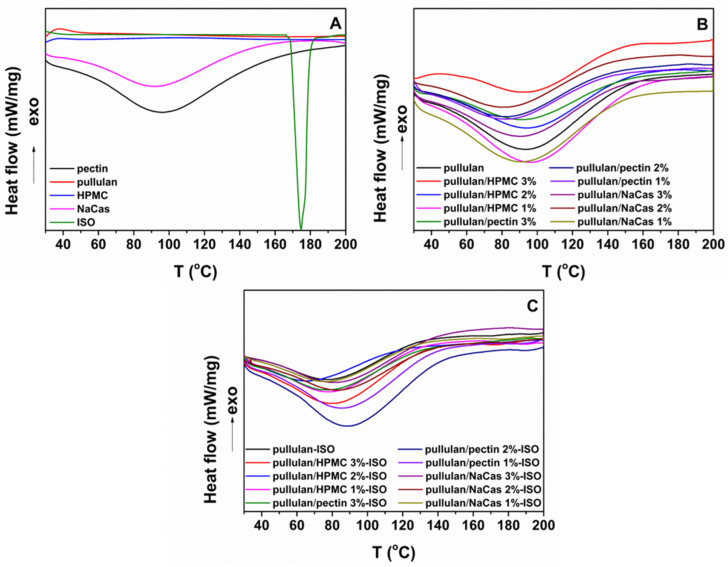
DSC thermograms of the (**A**) raw materials, (**B**) plain ODFs and (**C**) ISO-loaded ODFs.

The thermal properties of the raw materials and the plain and ISO-loaded ODFs were investigated using DSC analysis in order to assess the physical state of the drug in the fibrous formulations. Pure ISO (Figure 3A) demonstrated a sharp endothermic peak 174.9 °C, corresponding to the melting temperature of the crystalline drug, while the broad endothermic events observed in the excipients’ thermograms originated from moisture evaporation occurring due to their hygroscopic nature. The absence of the drug’s melting peak in the thermograms of the ISO-loaded ODFs (Figure 3C) indicates possible ISO amorphization, owing to the extremely fast solvent drying during the electrospinning process, which results in reduced drug mobility in the solidified polymer fiber matrix [40]. All ODF formulations demonstrated similar thermal profiles showing broad endothermic peaks attributed to water evaporation, as also verified during TGA analysis.

### 3.4. Thermogravimetric Analysis

**Figure 4:**
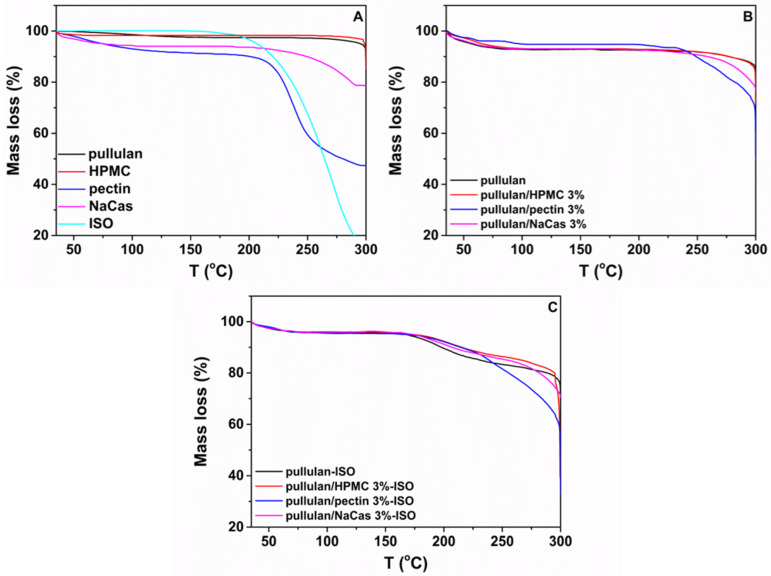
TGA curves of the (**A**) raw materials, (**B**) plain ODFs and (**C**) ISO-loaded ODFs.

Thermogravimetric analysis was carried out to investigate the thermal stability of the ODFs, and the thermograms of the raw materials and of representative ODFs are presented in Figure 4. All ODF formulations (Figure 4B) demonstrated two main thermal events. The first stage occurred at the temperature range of 40–100 °C and was attributed to the evaporation of the absorbed water, while the second thermal event was observed at temperatures above 250 °C for the pullulan and pullulan/HPMC 3%, attributed to the decomposition of the lower molar mass fractions of pullulan [41] and the cellulose ethers degradation of HPMC [42], respectively, at 232 °C for the pullulan/pectin 3%, associated with the depolymerization of pectin chains [43] and at ca. 170 °C for the pullulan/NaCas 3%, related to the degradation of NaCas [44]. The ISO-loaded ODFs were characterized by three stages of mass loss associated to moisture desorption (first), also showing an earlier onset of thermal decomposition (ca. 170 °C) compared to the plain ODFs, which corresponded to drug decomposition (second), followed by the biopolymers degradation (third). It is noteworthy that results indicated an acceptable thermal stability of the produced ODFs.

### 3.5. FTIR Analysis

**Figure 5:**
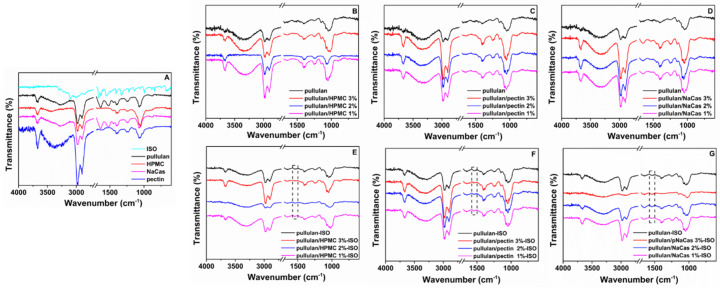
FTIR spectra of the (**A**) raw materials, the plain ODFs containing (**B**) HPMC, (**C**) pectin and (**D**) NaCas, and the ISO-loaded ODFs containing (**E**) HPMC, (**F**) pectin and (**G**) NaCas.

The FTIR spectra of the raw materials and the plain and ISO-loaded ODFs are shown in Figure 5. The characteristic peaks of pure ISO located at 3229, 3006, 1657, 1550 and at 1320 cm^−1^ can be attributed to the N–H stretching of amide groups, C–H symmetrical stretching, C=O stretching, N–H bending and NH_2_ waging vibrations, respectively [45]. HPMC showed a broad band at 3428 cm^−1^ assigned to the stretching frequency of the hydroxyl group, a band at 2981 cm^−1^ assigned to C–H stretching vibration and two bands at 1452 and 1378 cm^−1^ related to –CH_2_ scissoring and to the OH bending, respectively. In the spectrum of pectin, the peaks at 1727 and 1620 cm^−1^ are attributed to the esterified carboxyl groups and to the free carboxyl groups [46]. Pure NaCas showed a broad band at 3268 cm^−1^ assigned to the stretching of the N–H group. In the same spectrum, the peaks at 1637 and 1520 cm^−1^ corresponding to amide group I and to amide group II [43], could be attributed to the stretching of the carbonyl group –CO and to the symmetric stretching of N–CO bonds, respectively. Pullulan showed its characteristic bands located at 3268 (O–H), 2919 (C–H), 1637 (O–C–O) and 1394 cm^−1^ (C–O–H) [47] and a very sharp peak at 1054 cm^−1^ due to the stretching of the C–O–H bending vibration at the C-6 position [48], while its main characteristic peaks appeared to be the α–configuration of α–d-glucopyranose units at 845 cm^−1^ and the polysaccharide a(1,6) glycosidic linkage between glucose units at 931 cm^−1^.

In contrast to the plain ODFs, the FTIR spectra of the ISO-loaded ODFs demonstrated a peak at 1550 cm^−1^ attributed to amino group of ISO. It is worth noting that the peak at 1550 cm^−1^ was shifted to around 1542 cm^−1^ upon mixing with pullulan. The N–H vibration shifted to lower wavenumbers as pullulan concentration decreased (HPMC 3%: 1538, HPMC 2%: 1540, HPMC 1%: 1542 cm^−1^). This can be ascribed to the hydrogen bonding interaction developed between the hydroxyl groups of pullulan and the amino groups of ISO, in agreement with previous studies. [44] In addition, the change in the O–H peak intensity at 3660 cm^−1^ could also be related to hydrogen bond formation between pullulan and NaCas (Figure 5G), since a decrease in peak intensity at 3660 cm^−1^ is observed upon increasing NaCas concentration.

### 3.6. Disintegration Test

**Figure 6:**
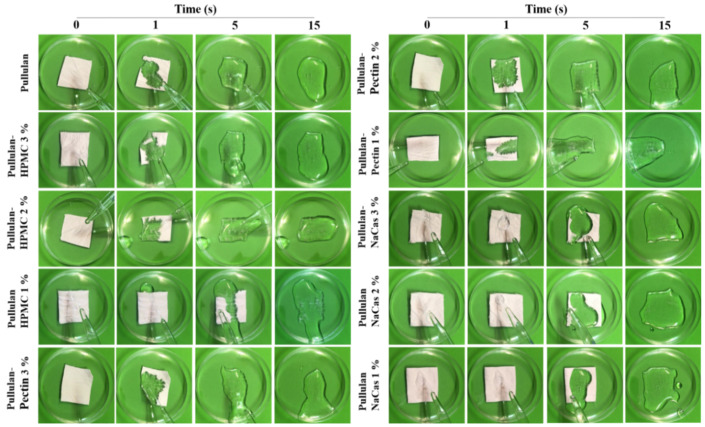
In vitro disintegration profiles of the ODFs in simulated salivary fluid as a function of time.

The disintegration profiles of the ODFs were recorded with a camera in SSF using the Petri dish set-up (Figure 6). It is clearly visible that complete disintegration of all ODF formulations occurred within less than 15 s. In particular, the inclusion of HPMC and pectin contributed to the rapid disintegration of the ODFs within 5 s, whereas the NaCas-containing ODFs showed a minor delay in the time required for their complete dissolution to occur compared to the rest of the tested formulations, which however, did not exceed 15 s. Hence, all drug-loaded nanofibers films instantaneously dissolved when brought in contact with the SSF medium, and the end-point of disintegration was approximately estimated to be 15 s. The disintegration time is an important quality attribute of ODFs; therefore, the complete dissolution of the prepared pullulan electrospun films containing HPMC, pectin and NaCas within less than 15 s meets the nonbinding recommendations of the FDA for drug products meant to be administered without chewing or liquids (<30 s) [49]. Representative videos from the disintegration profile of pullulan/HPMC 3% and pullulan/NaCas 3% are shown in Video S1 and S2, respectively.

### 3.7. In Vitro Drug Release in Simulated Salivary Fluid

**Figure 7:**
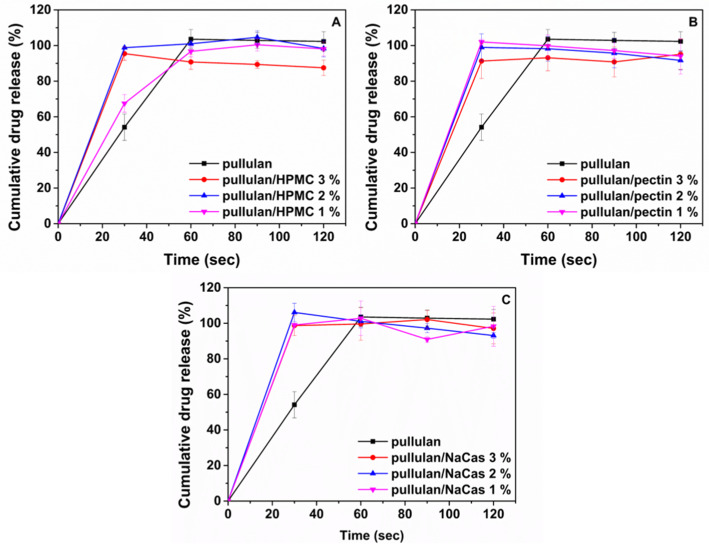
In vitro drug release profiles of ISO from the ODFs in simulated salivary fluid pH 7.0 at 37 °C.

In vitro drug release experiments were conducted in simulated salivary fluid pH 7.0 at 37 °C (Figure 7). All ODF formulations containing HPMC, pectin and NaCas demonstrated burst and total ISO release within 30 s, whereas plain pullulan ODFs released 54% of their drug content within the same timeframe, with complete drug release occurring at 60 s. The rapid ISO release from all ODF formulations could be attributed not only to the presence of the drug in an amorphous state within the nanofibers, as already shown with DSC analysis, but also to the unique properties of the electrospun nanofibers. In particular, the high surface to volume ratio of the ODFs significantly increases the contact area with the release medium, thereby accelerating ISO dissolution. In addition, the hygroscopic nature of HPMC, pectin and NaCas significantly enhance water uptake and fiber disintegration, thereby accelerating drug dissolution in the release medium. This might justify the relatively slower ISO release from the plain pullulan ODFs, due to the non-hygroscopic nature of pullulan [50].

## 4. Conclusions

Orodispersible films were fabricated as a child-friendly dosage form to facilitate oral drug administration to pediatric TB patients undergoing long-term isoniazid preventive therapy. With special attention drawn to the safety of the excipients chosen in pediatric formulations, the electrospun ODFs were fabricated from the aqueous solutions of natural and semi-synthetic polymers. To facilitate drug administration in a way that is as child-friendly as possible, the ODFs disintegrated instantly within less than 15 s upon contact with simulated salivary fluid, achieving rapid and complete ISO release within less than 60 s. Results indicate that all prepared ODF formulations constitute an age-appropriate dosage form for the pediatric population. Nevertheless, pullulan/HPMC-containing films could be considered a pertinent candidate for the development of ODFs based on their fast disintegration in simulated salivary conditions within less than 5 s, ensuring convenience of administration, and burst and complete ISO release achieved within 30 s, making the drug readily available for absorption in the oral cavity and across the gastrointestinal tract after swallowing. Further optimization of the ODF formulations with taste-masking excipients and in vivo taste and acceptability assessment studies are required to provide an effective therapeutic intervention for the pediatric population.

## Figures and Tables

**Table 1:** Shear viscosity and conductivity values of the spinning solutions.

Formulation	Shear Viscosity (Pa·s)	Conductivity (μS/cm)
Pullulan	2.09	65 ± 0.1
Pullulan/HPMC 3%	11.00	91 ± 0.2
Pullulan/HPMC 2%	5.63	85 ± 0.1
Pullulan/HPMC 1%	2.08	77.5 ± 0.5
Pullulan/pectin 3%	4.85	498 ± 1.0
Pullulan/pectin 2%	3.94	343 ± 0.6
Pullulan/pectin 1%	2.81	248 ± 2.0
Pullulan/NaCas 3%	1.39	385 ± 0.6
Pullulan/NaCas 2%	1.66	239 ± 0.6
Pullulan/NaCas 1%	2.01	192 ± 0.6

**Table 2:** Drug loading capacity and encapsulation efficiency of the ISO-loaded develop orodispersible films (ODFs).

Formulation	Loading Capacity (%) (± SD)	Encapsulation Efficiency (%) (± SD)
Pullulan-ISO	10.85 ± 0.57	90.42 ± 4.78
Pullulan/HPMC 3%-ISO	11.77 ± 0.13	98.11 ± 1.12
Pullulan/HPMC 2%-ISO	11.46 ± 0.20	95.52 ± 1.71
Pullulan/HPMC 1%-ISO	11.25 ± 0.79	93.80 ± 6.63
Pullulan/pectin 3%-ISO	11.45 ± 0.26	95.14 ± 2.2
Pullulan/pectin 2%-ISO	11.66 ± 0.32	97.20 ± 2.64
Pullulan/pectin 1%-ISO	11.28 ± 0.94	93.97 ± 7.84
Pullulan/NaCas 3%-ISO	11.40 ± 0.43	95.05 ± 3.59
Pullulan/NaCas 2%-ISO	11.06 ± 0.68	93.00 ± 5.66
Pullulan/NaCas 1%-ISO	11.13 ± 0.71	92.79 ± 5.94

## Supplementary Materials

The following are available online at https://www.mdpi.com/1999-4923/12/5/470/s1. Video S1: Disintegration test of pullulan-HPMC 3% ODF in simulated salivary fluid. Video S2: Disintegration test of pullulan-NaCas 3% ODF in simulated salivary fluid.
